# A Multi-Layer Feature Fusion Method for Few-Shot Image Classification

**DOI:** 10.3390/s23156880

**Published:** 2023-08-03

**Authors:** Jacó C. Gomes, Lurdineide de A. B. Borges, Díbio L. Borges

**Affiliations:** 1 Department of Mechanical Engineering, University of Brasília, Brasília 70910-900, DF, Brazil; cirino.jaco@gmail.com; 2 Embrapa Cerrados, BR-020, km 18, Planaltina 73310-970, DF, Brazil; lurd.borges@gmail.com; 3 Department of Computer Science, University of Brasília, Brasília 70910-900, DF, Brazil

**Keywords:** few-shot learning, metric learning, multi-layer feature fusion, multi-scale features, maize crop insect classification

## Abstract

In image classification, few-shot learning deals with recognizing visual categories from a few tagged examples. The degree of expressiveness of the encoded features in this scenario is a crucial question that needs to be addressed in the models being trained. Recent approaches have achieved encouraging results in improving few-shot models in deep learning, but designing a competitive and simple architecture is challenging, especially considering its requirement in many practical applications. This work proposes an improved few-shot model based on a multi-layer feature fusion (FMLF) method. The presented approach includes extended feature extraction and fusion mechanisms in the Convolutional Neural Network (CNN) backbone, as well as an effective metric to compute the divergences in the end. In order to evaluate the proposed method, a challenging visual classification problem, maize crop insect classification with specific pests and beneficial categories, is addressed, serving both as a test of our model and as a means to propose a novel dataset. Experiments were carried out to compare the results with ResNet50, VGG16, and MobileNetv2, used as feature extraction backbones, and the FMLF method demonstrated higher accuracy with fewer parameters. The proposed FMLF method improved accuracy scores by up to 3.62% in one-shot and 2.82% in five-shot classification tasks compared to a traditional backbone, which uses only global image features.

## 1. Introduction

Based on human-like learning, in which a few samples are sufficient for generalization, few-shot learning (FSL) [1] aims to optimize deep learning models by learning from just a few samples. This approach avoids the need for very complex models and also the huge amount of time spent on building large, labeled datasets. The meta-learning paradigm, as a strategy for cross-task learning, has been used in modern FSL models, achieving promising results in transfer learning [2,3,4]. Learn-to-measure, or metric-based, is an important method in meta-learning for obtaining prior knowledge from the similarities between images [5]. A typical learn-to-measure model is composed of a Convolutional Neural Network (CNN) image feature extractor and a similarity function for differentiating images in an embedding space. Some prominent models are prototypical [6], relation [7], and matching [8] networks, and their variations.

Some recent works have shown the good performance of these models by enhancing image representativeness. However, keeping an architecture simple to extract relevant image features is a challenge. In [9], an improved prototypical network, IPN, that combines an enhanced feature extractor and a distance-scaling strategy was proposed. The authors improved prototype representativeness by including intra-class information through a weight distribution module over the instances. Ref. [10] improved prototypical networks by combining recognition and object localization. Salient region detection was proposed for insect classification in complex-scenario images. The authors of [11] introduced FEAT (few-shot embedding adaptation with a Transformer) to adapt embeddings via a set-to-set function and a transformer architecture. FEAT improves discriminative feature extraction while yielding task-specific embeddings.

Although feature extraction improvement is promising, most of the works deal with recurrent similarity metrics such as the squared Euclidean distance [6,12], absolute difference (L1) [13,14], and cosine similarity [8,15]. Recently, these results have been improved by combining a feature extractor with a classifier based on a regularized Mahalanobis distance [16]. Improving the existing few-shot techniques for automatic classification is important for dealing with classes with high similarities between them, as in the case of crop insect pests, e.g., [17], in which the great similarities between species make the learning onerous. Our work here proposes to contribute to the advances in few-shot metric models by providing an improved backbone for image feature extraction and enhancing embedding similarity measurements with relative entropy.

Several areas have benefited from few-shot learning in visual recognition. Recent works have shown the great importance of this approach in agriculture. Li and Chao [18], for example, proposed a semi-supervised few-shot learning approach to solve plant-leaf disease recognition problems with fewer labeled training data. The authors of [19] reported a considerable reduction in training data with Siamese networks and a triplet loss for plant disease classification. Their model outperformed classical learning models on small training sets. In contrast, insect pest recognition is a critical case in agriculture because of the high potential for damage, and there are many similar insect species to differentiate. The similarities between insect species make automatic recognition one of the most challenging tasks in precision agriculture. Helping to mitigate this problem, the authors of [17] proposed a few-shot insect pest recognition model for specific maturity stages. The authors of [12] classified cotton crop pests using prototypical networks in an embedded terminal. Li and Yang [2] analyzed the cross-domain few-shot classification problem. They investigated insect and plant disease domains by changing and mixing them in meta-training and meta-testing datasets. Nuthalapati and Tunga [20] improved multi-domain few-shot learning in crops by updating the image embeddings using Transformers. Their model can capture important image features that are used to incorporate information about other support samples in a classification task. Cross-domain classification is a challenging task due to the limitations of the models in accumulating knowledge. As a solution to this problem, Li and Chao [21] proposed a continuous insect and plant disease classification model via the storage and retrieval of information from previous tasks. Their model combines Siamese networks and generative adversarial networks, which are used to extract and store information from tasks.

Advances in machine learning have made automatic insect recognition an approach used worldwide in agriculture. At present, deep learning is the most commonly used method, which has shown excellent performance, as demonstrated in many works [22,23,24]. On the other hand, deep learning models consume large amounts of data for training, which makes learning difficult since few public insect datasets are available in the literature, and building a new, large dataset is very time-consuming. Data augmentation [25] and transfer learning [26] are common approaches used to tackle the lack of training data. A recent review of research on insect classification can be found in [27]. Few-shot learning, however, could be a more suitable approach to addressing the challenging issue of insect crop classification in field images, as it can be used to design a lighter and more efficient model for this challenging scenario.

Maize is a major grain crop worldwide. With the progressive increase in global demand for this cereal, crop yield management is a factor that requires great attention to avoid economic losses. Intelligent technologies [28] are useful for the prior visual recognition of maize insects for decision making and mitigating damage to crops. However, most state-of-the-art machine learning models need high computing power and a large dataset to train algorithms, which are typically deep and complex, making it difficult to use this technology for on-site applications in agriculture.

Although recent studies have provided relevant results in the insect pest classification context, there is a gap in few-shot classifications of beneficial and non-beneficial insects. Beneficial insects have great importance in the biological control of pests that cause damage to crops. In this work, pests and beneficial insects in maize crops are included in a few-shot classification discussion.

This work introduces a multi-layer feature extractor and a similarity measurement approach for few-shot learning. Our method aims to improve image pattern recognition through multi-layer feature fusion, using a shallow CNN architecture to generate consistent visual representations and accurately measure similarities between images. Our model provides a promising development in few-shot insect classification using a dataset consisting mostly of field images. Furthermore, it can be easily adapted for other image pattern recognition applications.

The contributions of this work are threefold:

- A few-shot model based on multi-layer feature fusion to improve and combine the feature extraction process.
- The combined use of relative entropy to better distinguish the rich embeddings provided earlier in the fusion process.
- Testing and comparison, using a proposed maize crop insect (pest and beneficial) dataset, against consolidated backbones showing better performance, with less number of parameters, of the proposed model.

The rest of this paper is organized as follows. Section 2 provides details of the dataset built for a maize crop system and the proposed few-shot learning method and its parameters. Section 3 explains the experiments, Section 4 presents the results achieved, Section 5 discusses the results, and Section 6 presents the conclusions.

## 2. Materials and Methods

### 2.1. A Small Maize Crop Insect Dataset: Pests and Beneficial Insects

For this work, we gathered a very specific and challenging dataset for few-shot learning: maize crop insects, pests, and beneficial insects. Each crop has and attracts a set of possible pests and beneficial insects. Maize crops are one of the most important crops globally, and the automatic identification of insect species in these environments is of major interest. The dataset comprises 28 insect species, 17 insect pest classes, and 11 main natural enemies associated with maize pests [29]. The images are of a fixed size of 224 × 224 in RGB (red, green, blue), and they were selected from the internet and validated by the specialists consulted. For the few-shot scenario, we selected 15 images per class, totaling 420 images. All the images and the code are available at https://github.com/Jacocirino/Maize_crops_few-shot_insect_dataset.git (accessed on 19 July 2023).

**Figure 1:**
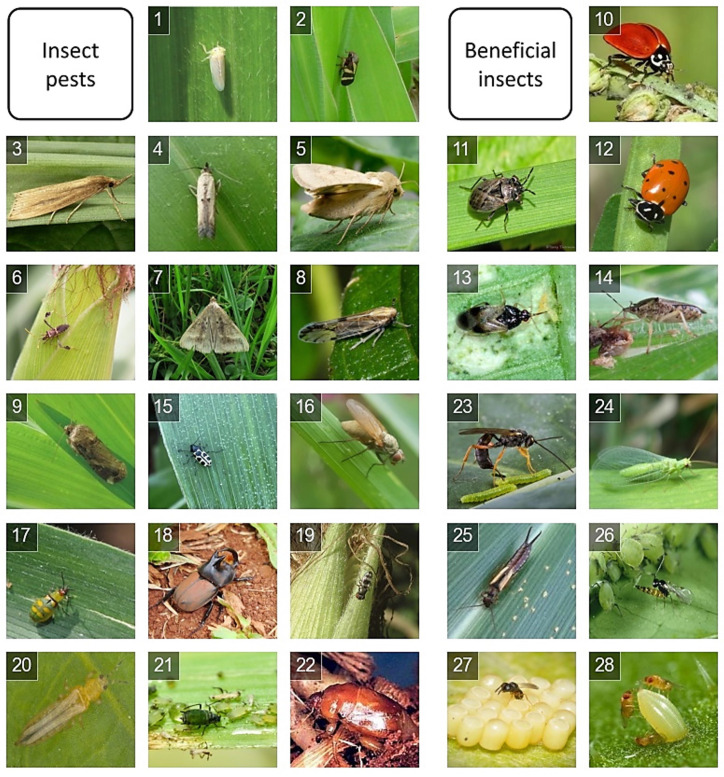
Sample images from each class in the dataset.

The specifications of this insect dataset are detailed in Table 1, which shows the species divided into source (also referred to as base data for training) and target datasets. These datasets are used here to meta-train and meta-test the proposed few-shot model, respectively. Figure 1 shows the classes of the data in two categories: pests and beneficial insects.

### 2.2. Few-Shot Learning

Few-shot learning refers to learning from a few data samples. It has received great attention in recent years due to deep learning advances. The most recent metric-based few-shot classifiers consist of two main branches: an image feature extractor functioning as an embedding function, and a similarity measurement block that operates within an embedding space. Some few-shot models learn embeddings to discriminate between pairs of images, such as Siamese networks [13] and triplet networks [30]. Basically, twin nets are used to extract image features, followed by a similarity measurement for classifying images within an embedding space. Multi-class models aim to perform something similar by classifying images among N classes, as performed in prototypical networks [6], relation networks [7], and matching networks [8], along with their variations.

**Figure 2:**
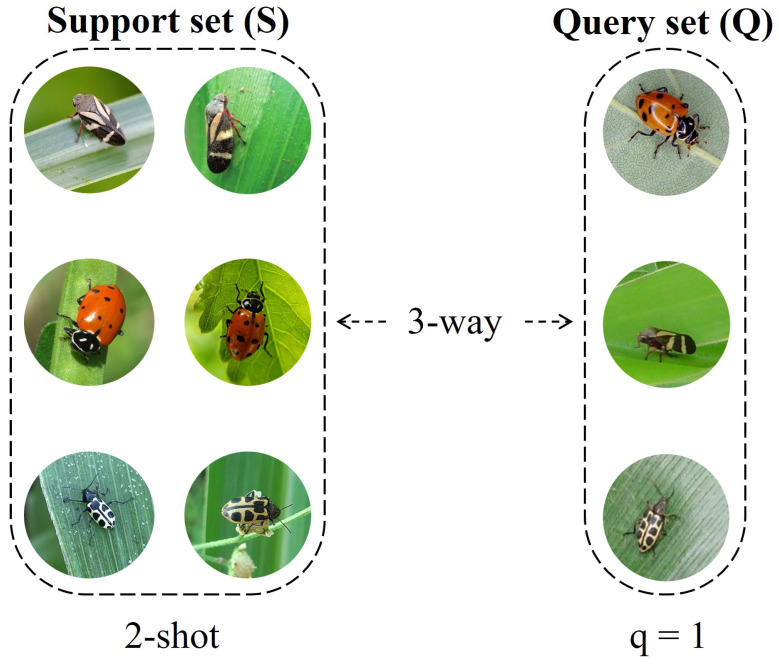
Sample sets in a few-shot classification task.

Multi-class few-shot models are typically trained through several N-way, K-shot classification tasks, in which there are N classes, each containing K support and q query samples per class. Also, the support and query sets within a task are commonly denoted as S = N ∗ K and Q = N ∗ q, respectively, as shown in Figure 2. Few-shot models aim to label the Q set images according to the N classes in a given task. The model learns from the source dataset (used as the base dataset for training, and divided into support and query sets) while classifying its query samples, and the training accuracy is computed. A test task is similar to a training task, except that the parameters of the model are frozen and classification is performed on the target dataset.

Our proposed method is based on the prototypical paradigm [6] and involves enhancing few-shot learning with an improved multi-layer extraction and fusion method. The method is detailed in Section 2.3.

### 2.3. The Proposed Method

A typical deep learning dataset usually contains thousands of images providing a significant amount of information about classes. In few-shot learning, however, there is little data available for constructing prior knowledge about categories. Determining how complex the features to extract are and how to use them is a challenging issue. Global image feature extraction is commonly used to create representative embeddings in most works involving few-shot learning [2,6,7,12]. However, when local features are neglected, important image information is lost and the effective few-shot similarity measure between embeddings can be penalized. Our proposed method is based on the hypothesis that fusing internal features from CNN layers is more efficient than using only global information. By fusing visual features, we expect to mitigate the loss of information for constructing candidates that strongly represent the categories. Moreover, we hypothesize that classifier performance can be enhanced in this scenario by using a divergence measure, such as relative entropy, instead of solely relying on a distance metric.

Multi-layer (or multi-scale) feature fusion is a promising method for extracting rich information from images and can improve model performance in deep learning [31,32]. The extraction of multi-scale features from different convolutional layers is a common method. However, how to utilize these features can define the complexity and performance of the model. Feature Pyramid Networks (FPN) [33], for example, attempt to fuse the features from multiple scales to predict bounding boxes for object detection. The authors of [34] proposed a feature fusion mechanism based on self-attention. Their approach includes a feature selection module for fusion in remote sensing few-shot image classification. The authors of [35] proposed multi-layer feature extraction for relation networks. They extracted multi-layer features from a CNN and generated relation features by calculating the absolute value of the difference between them. The authors of [36] extracted multi-scale semantic features using a pyramid structure and matched them to form multi-scale relation maps. Image classification is performed based on relation scores.

**Figure 3:**
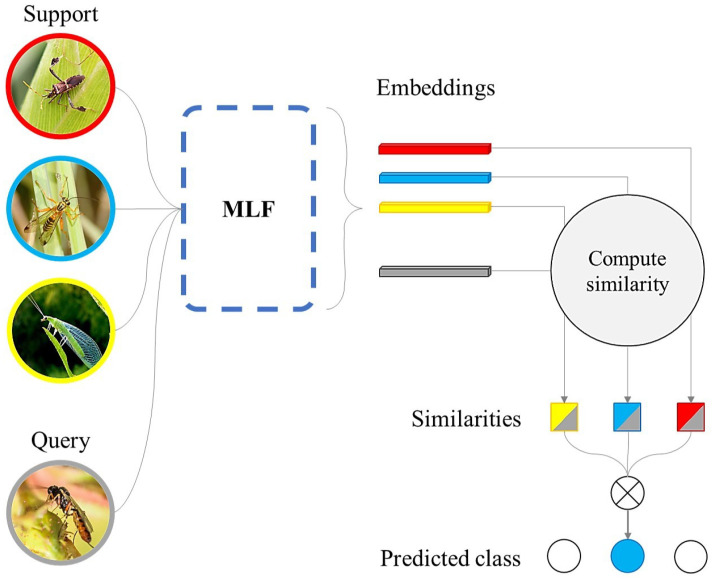
Framework of the proposed FMLF classifier using a 3-way, 1-shot setup with q = 1. Multi-layer features are extracted and fused within MLF, so the similarities between the query and support images are more precisely quantified.

Our approach integrates a specific method of multi-layer feature extraction into the few-shot model while maintaining a simple architecture, and proposes a simplified method for merging these features for generating information-rich class representatives. Figure 3 summarizes the proposed model, which is composed of two main modules: multi-layer feature fusion (MLF) and a classifier that can be based on the Kullback–Leibler divergence, also known as relative entropy, or the Euclidean distance. We have named this model FMLF (few-shot learning via multi-layer feature fusion).

Our MLF is a feature extractor based on a shallow backbone, which is used to form discriminative image representations. FMLF fuses low-level local, mid-level, and high-level global information from support and query images to form rich embeddings with class information. The comprehension of the workings behind the convolutional layers of a CNN was intuitively presented in [37]. The first layers of a CNN describe a detector of low-level features, such as edges. On the other hand, the deeper layers of the network detect high-level features, such as more defined objects or parts of them, in the images. Our proposal is to merge these resources from low to high levels to generate consistent vectors of information. The similarity module of FMLF computes the similarities among the rich embeddings in a task to perform image classification. The next sections detail each module of the FMLF, and implementation details follow.

#### 2.3.1. The Proposed Feature Extractor

Convolutional Neural Networks (CNN) are powerful methods for extracting low-, middle-, and high-level discriminative image features, even in shallower CNN architectures [31,38]. We propose enhancing a shallow backbone with a multi-layer feature fusion method for few-shot recognition.

**Figure 4:**
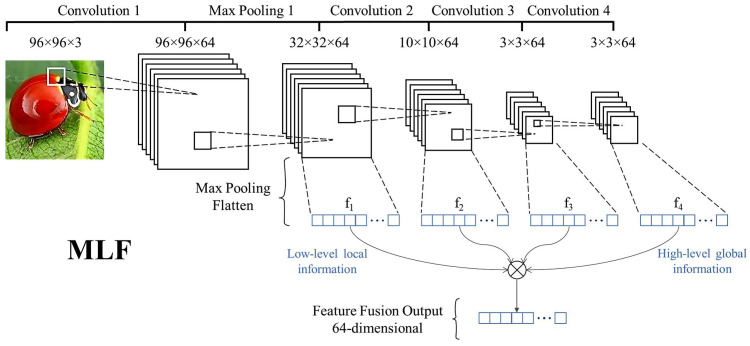
The proposed CNN multi-layer feature extraction and fusion (MLF) architecture.

The proposed CNN consists of a backbone with four main convolutional blocks, as depicted in Figure 4 (convolutions 1 to 4). Each block contains a convolutional layer with 64 3 × 3 filters, batch normalization, a Rectified Linear Unit (ReLU), and, finally, a 3 × 3 max pooling layer. The MaxPool output from the first block is shown in Figure 4 (Max Pooling 1), but it is suppressed from the subsequent blocks in the figure to avoid clutter. After each block output, MaxPool and Flatten operations are performed to generate 64-dimensional representation vectors (f${}_{1}$, f${}_{2}$, f${}_{3}$, f${}_{4}$), which are then averaged to produce the Feature Fusion Output (FFO). In other words, through a respective embedding function $f_{\phi }$ with learnable parameters ϕ, feature fusion computes the mean of the f-embedding representations from the layers in $f_{\phi }$, formulated as

(1) $${\mathrm{FFO}}^{n}=\frac{1}{4}\sum_{i=1}^{4}f_{i}\left(x\right),$$

where ${\mathrm{FFO}}^{n}$ represents the embedding of the image *x* from class *n*.

FFO provides rich embeddings for image representation. All data (support and query samples) in a task pass through the MLF architecture to create support and query embeddings. In order to obtain class representative vectors, prototype generation is performed on the support embeddings after the FFO stage. Each prototype corresponds to the centroid of the class embedding cluster, calculated as follows:

(2) $$c_{n}=\frac{1}{K_{n}}\sum_{i=1}^{K_{n}}{\mathrm{FFO}}^{n}\left(x_{i}\right),$$

where $c_{n}$ represents the centroid of K-shot from the class *n*. $K_{n}$ denotes the number of images labeled with class *n*.

MLF contributes to yielding prototypes rich in class information, as well as query embeddings rich in image feature information. Query image classification in a task is performed through similarity measurement between prototypes and query embeddings, as detailed in the next section and shown in Figure 3.

#### 2.3.2. The Proposed Similarity Measurement

Similarity measurement is crucial for differentiating images in a metric-based few-shot model. Some metrics are investigated to compare embeddings, such as $L_{1}$ [13,14], the squared Euclidean distance [6], cosine similarity [8], and Mahalanobis distance [16,20]. Although these metrics provide interesting results, other divergence methods are rarely investigated in few-shot learning. In addition to the Euclidean distance, which has already been adopted in many works, relative entropy is adopted as a similarity function in this work to enhance the comparison of embeddings.

The Kullback–Leibler divergence, also called relative entropy, is a Bregman divergence widely used in signal processing. It computes a score that quantifies the divergence between two probability distributions, which is defined in terms of a strictly convex function based on entropy [39], and is expressed as follows:

(3) $$\mathrm{KL}\left(x∥y\right)=\sum_{j=1}^{d}x_{j}log_{2}\left(\frac{x_{j}}{y_{j}}\right),$$

where *d* denotes the size of the distributions.

The logarithm can be base-2 or base-e to measure divergence in units of bits or nats, respectively. Relative entropy is not considered a distance metric since it is asymmetric (i.e., KL(x∥y)≠KL(y∥x)) and does not satisfy the triangle inequality. To compute relative entropy in this work, probability vectors are generated from all task instances, ensuring that s=s/sum(s) for each prototype and query embedding. Additionally, $\sum_{i=1}^{d}s_{i}=1$. To avoid infinitely negative results caused by log0 and division by zero, a small value ϵ was added to the embeddings just before the relative entropy computation in (3).

#### 2.3.3. Implementation Details

The feature extractor in FMLF inputs images resized to a 96 × 96 × 3 format and outputs 64-dimensional embeddings. All images in the dataset were rotated by 90°, 180°, and 270° to increase the initial number of classes 4-fold.

The variation of the N-way and K-shot parameters can be approached in different ways [2,3,18,20]. In this work, these parameters, as well as the q query samples for the training and testing tasks, are set to N=5, K=1 and 5, and q=5. Query images are labeled according to a probability distribution based on divergences. A divergence value close to zero means that two distributions are very similar and have a high probability of belonging to the same class. Conversely, as the divergence value increases positively away from zero, it means that the difference is high, and the probability is low. The probabilities in a task are determined by a softmax function, formulated as follows:

(4) $$p_{\phi }\left(y=n|x\right)=\frac{exp(-d\left(f_{\phi }\left(x\right),c_{n}\right))}{\sum_{n^{\prime }}exp\left(-d\left(f_{\phi }\left(x\right),c_{n}^{\prime }\right)\right)},$$

where *d* denotes a distance or comparative function.

We used the Adam optimizer to minimize the negative log-probability $J\left(\phi \right)=-log_{\phi }\left(y=n|x\right)$ of the true class *n*.

## 3. Experimental Setup

A set of experiments was conducted to analyze the behavior of the proposed model in classifying maize crop insects. The experiments consisted of (1) training and testing the model by extracting only global features ($f_{4}$ backbone), (2) training and testing the proposed FMLF, and (3) evaluating three different backbones from the literature as feature extractors for result comparison—ResNet50 [40], VGG16 [41], and MobileNetv2 [42]—with their fully connected layers (FC) removed. Table 2 presents the total number of parameters in each model, including ours. All experiments were performed using both divergences: relative entropy (RE) and the Euclidean distance (ED). The same N-way, K-shot, and dataset configurations, as previously described, were used across all experiments.

Experiments were conducted using the Google Colaboratory platform. In addition, the Pytorch library version 1.12.0 and a Tesla T4 GPU were used to construct and train the models. During training, the source dataset was used to train the models as the base dataset over 100 epochs, each comprising 150 episodes (an episode denotes a classification task). The learning rate, initially set to $10^{-3}$, was halved after 20 epochs. When testing, the target dataset was used to classify the images. The performances of the models are presented here as the average accuracy across two thousand testing episodes.

## 4. Results

Our experiments focused on differentiating and classifying pests and beneficial insects in maize crops. An insect dataset was built, and we divided it into source and target datasets. As shown in Table 1, there was no overlap between the classes in the source and target datasets. Furthermore, each class contained only 15 images for few-shot classification.

In order to evaluate the performance of the proposed model, comparisons were conducted regarding the backbones and divergences. Firstly, we trained and tested the backbone with only its global feature (the $f_{4}$ backbone, see Figure 4 for more details about the f${}_{4}$ backbone). In addition, we enhanced it using our MLF method to extract and fuse local, middle, and global information for classifying images in a rich embedding space. Our analysis considered both one-shot and five-shot tasks and Table 3 shows the results using the two investigated divergences.

Few-shot learning models are typically evaluated through a set of classification tasks. Each entry in Table 3 corresponds to the mean of 2000 test tasks on the target dataset.

Finally, our approach was compared to other backbones, and these results are presented graphically in the discussion.

## 5. Discussion

Learning from a few samples is a challenging task in visual recognition. In few-shot classification, extracting rich information vectors from images and obtaining accurate similarity measurements between classes, especially in situations with very similar objects, are important factors in classification performance. Improving these aspects of the models is a promising approach for enhancing few-shot insect recognition in agriculture since the similarities between insect species make classification a challenging task. Moreover, accurately differentiating between pests and beneficial insects presents a promising approach for avoiding crop losses and unnecessary pesticide applications, as it can contribute to biological insect pest control. In this work, our FMLF model was able to improve few-shot classification in two respects: by better utilizing the CNN architecture to extract rich information vectors through multi-level image feature fusion, and by effectively employing relative entropy and the Euclidean distance to improve embedding comparison.

According to the results in Table 3, the $f_{4}$ backbone, which used only the global features, provided better results, with accuracies of 66.22% and 78.82% in the one-shot and five-shot scenarios, respectively. These results were improved with the fusion of multi-layer features using the MLF extractor. Our FMLF method improved the accuracy of the model’s performance by up to 3.62% in the one-shot scenario using relative entropy (RE), with an accuracy of 69.84%, and 2.66% in the five-shot scenario using the Euclidean distance (ED), with an accuracy of 80.82%. These results show that multi-level feature fusion is a promising approach for enhancing the results of few-shot insect classification, especially using limited data like the one-shot scenario, where the gain was more pronounced.

**Figure 5:**
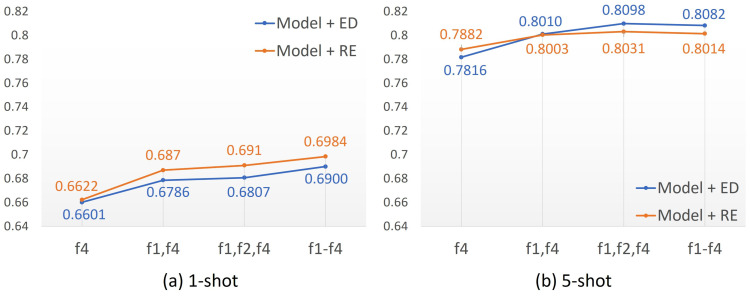
Accuracy trends as f-features are fused.

The $f_{4}$ backbone was slightly enhanced with RE compared to the ED in both the one-shot and five-shot scenarios. However, RE outperformed the ED in the one-shot scenario, whereas the ED achieved better results in the five-shot scenario using the MLF method. It is not possible to conclude which divergence was the best; only that they provided slightly different results from a K-shot perspective. For example, in the one-shot scenario, RE appears to be a promising approach, as can be seen in Figure 5a. However, its results tended to be lower than those of the ED in the five-shot scenario, as shown in Figure 5b. Regardless of the best divergence method, Figure 5 shows that both divergences exhibited a positive sensibility in classifying the true class as the feature vectors were enriched with multi-layer information.

As demonstrated in Figure 5, accuracy tended to increase when the local f${}_{1}$ and global f${}_{4}$ features from the layers were fused (see Figure 4 for more details about f-features). The reason is that the FFO function yielded a more representative embedding when local information was fused with global information. It was observed that the model leveraged accuracy as more f-features were fused with global features in the one-shot scenario. On the other hand, in the five-shot scenario, the accuracies degraded slightly when using all f-features, with better performance achieved by merging the f${}_{1}$, f${}_{2}$, and f${}_{4}$ feature layers. This demonstrates the significant relevance of feature fusion for low-shot applications, such as one-shot, since much information is required for learning about categories.

**Figure 6:**
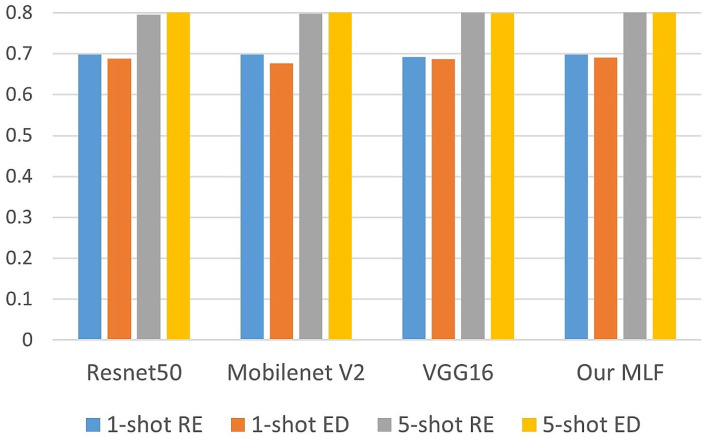
Performance of the models.

Our proposed method was also compared with the well-established backbones from the literature. Figure 6 shows the performance of the models concerning the K-shot and the divergence adopted. It can be observed that the Euclidean distance was the most suitable metric for the five-shot scenario, except for the VGG16 network. On the other hand, Relative entropy outperformed the ED for all backbones tested in the one-shot scenario.

**Figure 7:**
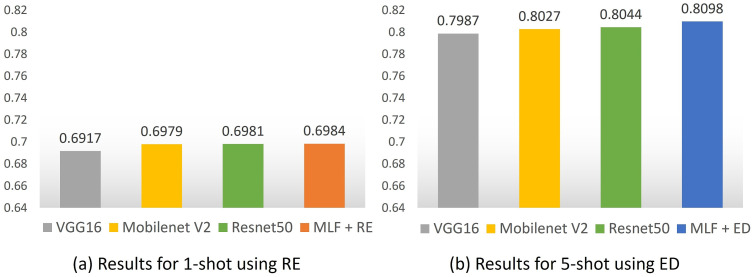
Best performance of the models. RE: Relative entropy. ED: Euclidean distance.

For the purpose of closely comparing the backbone performances, we combined the best accuracy results from each of the models. For the one-shot scenario, we evaluated the results using relative entropy, and for the five-shot scenario, we used the Euclidean distance. Figure 7 shows a comparison of our results with those of the Resnet50, VGG16, and Mobilenetv2 backbones, demonstrating the advantages of FMLF.

Although the results related to the backbones are approximate and could change slightly due to the few-shot classification task dependencies, our model contains a very small number of learnable parameters, as shown in Table 2. FMLF has 93% fewer parameters compared to Mobilenetv2 and 99% fewer parameters compared to ResNet50, the two most comparable backbones to our results. This makes FMLF a quick alternative for the application of insect recognition in crops using few samples.

The overall results showed that our model improved traditional global feature extraction, achieving improvements of up to 3.62% in the one-shot scenario and 2.82% in the five-shot scenario, considering the best accuracies for the KL and ED, respectively. FMLF improved few-shot classification and demonstrated its ability to differentiate pests and beneficial insects from maize crops with relevant accuracy. It is important to note that the few-shot parameters were fixed throughout the experiments to analyze the results. We believe that even better results can be achieved by further adjusting these parameters, such as increasing the K-shot, which can improve accuracy because it increases the class representativeness reflected in the prototypes.

Considering the area of insect classification (both pests and beneficial insects) for agriculture, we propose that FSL approaches, such as this one, are an important avenue to be explored. Focusing on the most important (pests and beneficial) insect species in one crop system only, and yet tuning the approach for each other crop, could be more feasible in terms of hardware and accuracy considerations. Providing an integrated solution for each crop could even be more sustainable by avoiding insecticide spraying if the identification and counting of species could reach satisfactory levels.

## 6. Conclusions

This work proposed an improved few-shot classifier, named FMLF, which was tested in differentiating pests and beneficial insects from maize crops. The novelty mainly lies in extracting richer features and embeddings and fusing them in a multi-layer process within a few-shot setting. Experiments were conducted using a maize crop insect dataset containing both pests and beneficial insects, with 15 images per class. The results showed that our model (FMLF) achieved an accuracy of 69.84% in the one-shot scenario and 80.98% in the five-shot scenario, outperforming traditional feature extractors that use global feature extraction by up to 3.62% and 2.82% in one-shot and five-shot scenarios, respectively. Furthermore, our model outperformed the state-of-art backbones, Resnet50, VGG16, and Mobilenetv2, which were used here for feature extraction. These figures demonstrate the advantages of the proposed model in handling a challenging and interesting dataset. Reproducibility in other datasets may not be possible, and extending and testing this model to other settings will be considered in the near future. Few-shot learning and insect crop classification can be mutually advantageous when researched together since insect crop classification is a challenging visual task that requires fast and accurate solutions to be deployed in field crops.

## Figures and Tables

**Table 1:** Maize crop insect dataset classes.

	Source Set		Target Set
**Label**	**Insect Name**	**Label**	**Insect Name**
1	*Dalbulus maidis*	15	*Astylus variegatus*
2	*Deois flavopicta*	16	*Delia* spp.
3	*Diatraea saccharalis*	17	*Diabrotica speciosa*
4	*Elasmopalpus lignosellus*	18	*Diloboderus abderus*
5	*Helicoverpa zea*	19	*Euxesta* spp.
6	*Leptoglossus zonatus*	20	*Frankliniella williamsi*
7	*Mocis latipes*	21	*Rhopalosiphum maidis*
8	*Peregrinus maidis*	22	*Scaptocoris castanea*
9	*Spodoptera frugiperda*	23	*Campoletis flavicincta*
10	*Cycloneda sanguinea*	24	*Ceraeochrysa* sp.
11	*Geocoris* sp.	25	*Doru luteipes*
12	*Hippodamia convergens*	26	*Lysiphelus testaceipes*
13	*Orius* sp.	27	*Telenomus remus*
14	*Podisus* sp.	28	*Trichogramma pretiosum*

**Table 2:** Number of parameters in each backbone used for feature extraction.

Backbone	Parameters ($10^{3}$)
VGG16	138,365.99
Resnet50	25,557.03
Mobilenetv2	3504.87
f${}_{1}$ branch of MLF	1.92
f${}_{2}$ branch of MLF	38.98
f${}_{3}$ branch of MLF	76.03
f${}_{4}$ branch of MLF	113.09
Total of MLF	230.02

**Table 3:** Average accuracy of each model. ED: Euclidean distance. RE: Relative entropy.

Resource	1-Shot	5-Shot
$f_{4}$ backbone+ ED	0.6601	0.7816
$f_{4}$ backbone + RE	0.6622	0.7882
MLF + ED	0.69	**0.8082**
MLF + RE	**0.6984**	0.8014

## Data Availability

The code and dataset used during the current study are available at https://github.com/Jacocirino/Maize_crops_few-shot_insect_dataset.git (accessed on 19 July 2023).
